# Sincerity Is in the Eye of the Beholder: Using Eye Tracking to Understand How Victims Interpret an Offender’s Apology in a Simulation of Victim–Offender Mediation

**DOI:** 10.3389/fpsyg.2020.00835

**Published:** 2020-05-20

**Authors:** Florian Bonensteffen, Sven Zebel, Ellen Giebels

**Affiliations:** Faculty of Behavioral, Management and Social Sciences, Department of Psychology of Conflict, Risk and Safety, University of Twente, Enschede, Netherlands

**Keywords:** victim–offender mediation, apology, sincerity, visual attention, eye tracking, perceived responsibility taking, perceived suffering, offender resocialization attitudes

## Abstract

The objective of this study was to gain insights into how victims use their visual attention to determine the sincerity of an offender’s apology during simulated victim–offender mediation. We hypothesized that the victims’ visual attention (gaze fixation duration) would be focused more on the offender’s upper (than lower) face area, especially the eyes and the eyebrows, to infer the degree to which the offender suffers, takes responsibility, and has empathy for the victim. In turn, we expected these inferences to positively predict the perceived sincerity of the apology. Additionally, we took into account the victims’ *a priori* expectations regarding the sincerity of the apology and (positive) attitudes toward resocialization programs (ARPs). We expected both variables to enhance the above proposed process through which victims determine the sincerity of the apology. Fifty-eight students took the victim’s role in a fictitious crime scenario and watched a video in which the offender offered a remorseful apology. We obtained eye tracking data to determine the participants’ fixation and attention distribution. As expected, the participants’ gaze fixated significantly longer on the upper face. The results also showed that their prior expectations, positive ARPs, and inferences of suffering and responsibility taking after the apology all positively predicted the perceived sincerity. However, unexpectedly, gaze duration was not directly associated with these inferences. The fixation duration on the upper face instead appeared to moderate how ARPs predicted inferences of responsibility taking. More concretely, the exploratory path model analyses revealed that when the participants had more positive *a priori* ARPs, the longer they focused on the offender’s eyes and eyebrows and the more they concluded that he took responsibility for his actions (which in turn predicted *more* sincerity). However, for those with relatively negative ARPs, it was the other way around: the more they focused on the eyes and the eyebrows, the stronger they inferred that the offender did *not* take responsibility (which predicted *less* sincerity). Our findings demonstrate the vital role of the victims’ *a priori* attitudes, expectations, and eye gaze behavior in understanding the reception and the evaluation of offenders’ apologies. This study also suggests how novel technology can be used to investigate gaze behavior in the field of victim–offender mediation.

## Introduction

As an addition or even alternative to traditional punitive justice systems, contemporary restorative justice policies and practices have had a tremendous effect on humanizing court processes in Western societies (Walgrave, 2004; Shapland et al., 2006; Wenzel et al., 2008; Choi et al., 2010; Hansen and Umbreit, 2018). Among the most-established formal forms of restorative justice around the world, victim–offender mediation (VOM) engages those who were directly involved in the crime in a constructive dialogue in order to reach mutual, (im)material reparation (Umbreit, 2001; Umbreit et al., 2004; Shapland et al., 2008; Gromet and Darley, 2011; Johnstone and Brennan, 2014; Rypi, 2017; Hansen and Umbreit, 2018). Through its dialogue-driven nature, VOM directly involves victim and offender in order to facilitate an agreement about what the offender appropriately should do to repair the harm he or she caused (Bradshaw and Umbreit, 2003; Gromet and Darley, 2011), with the aim to make amends for the victim’s material and/or emotional pain (Weitekamp, 1993; Bradshaw et al., 2006; Choi and Gilbert, 2010). For victims, it is generally assumed that when they feel that they received a genuine apology as a form of symbolic restitution, this is one of the most important and helpful means of compensation (Nugent et al., 2001; Umbreit et al., 2005; Choi and Severson, 2009). More broadly, evaluations of VOM programs corroborate that the victims perceive the apology of the offender as one of the most important outcomes (e.g., Strang et al., 2006; Bolitho, 2012; Dhami, 2012, Dhami, 2016b). However, from a practical perspective, the victims’ individual evaluations of apologies received in VOM vary substantially, consequently leading to dissatisfaction with the mediation if they perceive the apology to be incomplete or insincere (e.g., Choi and Gilbert, 2010; Hansen and Umbreit, 2018). Even more so, rejected apologies are generally considered as undesirable as they promote the offenders’ negative feelings toward the victim, such as higher feelings of anger (Dhami, 2016b). An important question therefore is what underlying psychological processes may explain the victims’ positive or negative evaluation of apologies in VOM, yet surprisingly little is known about the emotional and perceptual dynamics within the restorative process and the way the victims come to their conclusions (Choi et al., 2010).

The current study aims to increase our understanding of the factors promoting or hindering the positive reception of an apology of the offender among victims. Specifically, it makes three contributions. First, based on what we know from the literature and mediation practice, we aim to model the interplay of distinct variables contributing to the victims’ perception that the offender’s apology is sincere. Second, the current study intends to answer the question to what extent it is possible to predict where the victims gaze at to draw inferences about the offender who offers his apology during VOM. Third, commonly utilized measurement instruments, such as interviews and questionnaires, come with tight restrictions as to their capacity to depict processes that are beyond consciousness. At the same time, the technological development over the last years provides more efficient and accurate methodology that is already applied in the social sciences (e.g., Newman et al., 2012). In VOM, the application of novel technology is in its preliminary stage. In various psychological contexts, eye tracking technology is used as a highly accurate, non-invasive means to determine the individuals’ attention distribution that is largely unconscious (e.g., Blechert et al., 2009; Buckner et al., 2010; Hall et al., 2015; Menon et al., 2016; Vraga et al., 2016; Frick and Pavlou, 2018; Frost-Karlsson et al., 2019). We want to ascertain how research into VOM might similarly benefit from the application of novel technology by introducing eye tracking to this field.

### Restorative Justice in Practice: Victim Offender Mediation

Traditional court processes in the Western society usually entail punitive responses to the wrongdoer’s deed, according to principles proposed by basic law constitution (Wenzel et al., 2008; Gromet and Darley, 2011; Gerkin et al., 2017). Critically, victims and offenders as those who are directly involved in the crime often face a lack of engagement corresponding with neglected needs in this process (Wenzel et al., 2008; Choi and Severson, 2009; Umbreit and Armour, 2010; Dhami, 2012). Therefore, alternative practices have been applied in the justice system in the last decades, referred to as restorative justice (RJ), to facilitate an understanding about reparation agreements (e.g., Walgrave, 2004; Latimer et al., 2005; Wenzel et al., 2008; Choi and Gilbert, 2010; Umbreit and Armour, 2010; Bolívar, 2013; Dhami, 2017; Hansen and Umbreit, 2018). With over a thousand programs in more than 20 countries, VOM has become one of the best-known and most-accessed professional initiatives of RJ practice worldwide (e.g., Nugent et al., 2001; Umbreit et al., 2004; Johnstone and Brennan, 2014; Freitas and Palermo, 2016; Hansen and Umbreit, 2018). It assists victims and offenders to share their narratives in a safe setting and under the guidance of one or more trained mediator(s) (e.g., Umbreit et al., 2004; Hansen and Umbreit, 2018). By this, both parties can engage in a constructive dialogue to, for instance, explain how the crime affected their lives, to ask questions, to apologize, and to acknowledge responsibility (e.g., Hansen and Umbreit, 2018).

VOM can occur via direct and/or indirect formats (McGarrell and Hipple, 2007; Choi and Severson, 2009). Direct mediation enables the victim and the offender to communicate face to face; common forms of indirect mediation encompass letter exchange between both parties and shuttle mediation, whereby a mediator relays messages between both parties if both parties can or do not want to meet directly (Freitas and Palermo, 2016). Direct, face-to-face mediation is commonly regarded to have a higher potential to avoid miscommunication in both parties due to the presence of non-verbal cues (Choi and Severson, 2009). It provides additional vocal and visual input to the victim from verbal and non-verbal cues that were reported to strongly impact the victims’ appraisal of the offender’s trustworthiness (Choi and Severson, 2009). For instance, Shapland et al. (2008) found that the victims are less likely to accept an offender’s apology when they do not see the offender during indirect mediation compared to direct VOM. Nonetheless, also in face-to-face mediation, the victims may reject an apology from the offender (Daly, 2013). This rejection is likely to impact the offender as well. In two experiments among university students, Dhami (2016b) found that offenders who faced rejection had more negative feelings of anger toward the victim, whereas those whose apology was accepted were more likely to reach an agreement with the victim (Dhami, 2016b).

### The Perceived Sincerity of an Apology

A substantial body of research indicates that, in order for an apology to be considered as complete, it should contain an acknowledgment of the wrongful act and an expression of regret for the harm it has caused and one should take responsibility for one’s wrongful behavior and outcomes. In addition, one should attempt to make amends and offer to repair the harm inflicted in order to account commitment for the negative consequences of the deed. Finally, a full apology also consists of promises not to repeat the behavior in the future (e.g., Choi and Severson, 2009; Slocum et al., 2011; Dhami, 2016a). Although distinct in terms of content, Dhami (2017) notes that these aspects may be interlinked in apology expressions so that a clear identification of each of these aspects in apologies might not always be possible (Dhami, 2017). Offering a complete apology can have positive effects on the person who receives it and in turn on the apologizer as well (Shnabel and Nadler, 2008, 2015). In their *Needs-Based Model of Reconciliation*, Shnabel and Nadler postulate that receiving an apology from the offender can positively affect the victim’s feelings of strength such as power, influence, and self-esteem, subsumed under the *agency need* dimension of the victim. An important requirement to fulfill such agency needs among victims, however, is that the message of the offender is perceived as sincere and conveying his or her true feelings of remorse (Choi and Severson, 2009).

A crucial question therefore is how and when the victims come to perceive an apology as sincere. Importantly^1^, proposed that the victims base their perceptions of sincerity on two psychological inferences that they may draw from a remorseful apologetic statement. First, the victims are tuned in into the level of internal suffering that the offender conveys through the statement as this indicates whether the offender considers the deed morally unjust and is affected by it emotionally. As^1^ point out, high levels of suffering will signal that the offender has a moral conscience and therefore s/he may be considered unlikely to repeat the offense in the future. However, showing suffering alone will not be satisfactory for the victims when the offender does not explicitly acknowledge having committed the offense. Therefore, the victims will be focused as well on the degree to which the offender takes responsibility for the offense. When the victims infer from the apology that the offender clearly takes the blame, they will consider this as a sign that the offender feels compelled to deal with and repair the harm inflicted. Accordingly^1^, demonstrated empirically that both inferences underlie how sincere the victims perceive the apology of the offender to be. In addition to this, based on their analyses of the offenders’ deliverance of apologies during VOM cases, Choi and Severson (2009) concluded that an apology must also convey that the offender empathizes with the victim to be considered as genuine and sincere. The current study aims to replicate and build on these findings, and therefore we propose the first hypothesis:

The victims’ inferences of suffering, responsibility taking, and degree of empathy for the victim in the offender’s apology jointly positively predict the perceived sincerity of the apology (H1).

### Where Do Victims Look at to Draw Inferences That Inform the Perceived Sincerity of the Apology?

The question arises of how the victims mentally process the visual and emotional expressions given by the offender or, in other words, convert the visible information into meaningful emotional attributions. To process information, the brain interacts with the environment by sending and receiving stimuli within the optic nerves and several brain regions (Toh et al., 2011). The brain is able to create a meaningful representation of the signals the eye receives from the surrounding environment. By shifting the focus of visual attention, also referred to as eye movement, individuals are able to interact with their social environment and to identify visible emotions in others. Consequently, we are able to compound several individual visual stimuli into a meaningful whole in order to form attributions about others, rooted in the conceptions of Gestalt theory (Toh et al., 2011). Literature agrees that the face is considered to be a major source of social information about a person, such as familiarity or emotional and mental state (e.g., Smith et al., 2005; Chaby et al., 2017; Itier and Neath-Tavares, 2017). Facial emotion recognition theory, pioneered by the American anthropologist Paul Ekman, suggests that a set of primary or basic emotions expressing *happiness*, *surprise*, *anger*, *sadness*, *fear*, and *disgust* (*contempt* was later added as the 7^*th*^ basic emotion directly recognizable in the human face) can directly be expressed through the contraction of certain facial muscles (e.g., Ekman and Cordaro, 2011). These basic emotions are largely considered to be universal across different cultures around the world (Ekman and Cordaro, 2011, but see also Sabini and Silver, 2005; Sato et al., 2019). In the same vein, Adolphs (2003) concludes that facial displays of emotions are direct indications of the intentions or the moods of the subject, thus indicating that there might be a direct relation of inner emotions and facial expressions. Ekman and Friesen (1978) earlier introduced the Facial Action Coding System (FACS) as a means to denote facial muscle contraction into meaningful interpretations of emotions, which has been developed further in later years by Ekman and colleagues. The model proposes that facial expressions consist of smaller components related to minimalistic impulses on one or more facial muscles called action units (AUs). According to the model, 44 AUs related to certain facial muscles exist; these are listed numerically in the coding scheme. For instance, expressing sadness activates three AUs (*inner brow raiser*, *brow lowerer*, and the *lip corner depressor*) that are contracted to create a facial expression that is congruent with the emotional state. Accordingly, literature agrees that particular face regions rather than others have a higher discriminating potential to express a specific emotion that is associated with muscle contractions in this region (Smith et al., 2005; Chaby et al., 2017). In a study among adults, Chaby et al. (2017) tested gaze behavior consistency when the participants were exposed to emotional faces. Interestingly, when faces express various basic emotions, there was a difference in the participants’ attention fixation on the facial areas, also called areas of interest (AOIs). In particular, the results indicated differences in attention distribution among the AOI of the lower and the upper face. When looking at faces that expressed joy and disgust, more fixations were detected on the lower part of the face, which included all facial areas down from the tip of the nose, including the mouth and the chin. In contrast, when faces expressing the emotions of fear, anger, and sadness were shown, attention was directed to the upper part of the face, including the eyes, the eyebrows, and the forehead (Chaby et al., 2017); similar findings were presented earlier by Calder et al. (2000).

An exact allocation of the emotional inferences we predict to be associated with sincere apology, suffering, responsibility taking, and empathy for the victim in one of the predefined areas, however, seems challenging when taking existing literature into consideration. Thus, we suggest a more abstract categorization based on familiar and related emotions the victims may perceive during an apology (see also Gerrod Parrott, 2010). ^1^Have shown that common emotions that might be used during an apology (shame, guilt, regret, and sadness) all communicate levels of suffering and responsibility taking to their recipients, but to a different degree (with highest levels being inferred from guilt and shame). Therefore, we assume that the inferences of *suffering* and *responsibility taking* drawn from (the emotions conveyed during) a remorseful apology are likely to be derived from the same facial region where the emotion sadness is located: the upper face area (Calder et al., 2000; Chaby et al., 2017). Hence, the following hypotheses are formulated:

During the observation of the offender who gives a remorseful apology, the victim’s attention is focused more on the offender’s upper face area than on the lower face area (H2).

Moreover, we expect that the degree of eye gaze fixations on the offender’s upper face part positively predicts the perceived suffering, responsibility taking, and empathy inferences, which in turn contributes to the perceived sincerity of the apology (H3).

### Taking Into Account the Victims’ Expected Sincerity and Their Attitudes Toward Resocialization

Of course, the victims will differ in terms of the expectations and attitudes that forego their engagement in a VOM program (Karliczek et al., 2013; Hansen and Umbreit, 2018). It is inevitable to take into account the victims’ prior expectations regarding the sincerity of the apology (henceforth, expected sincerity) when examining the perceived sincerity of the apology as a desired outcome of VOM (Bolívar, 2013; Dhami, 2016a). Along the same line, several authors have argued and empirically demonstrated that the motivation to contribute to the offender’s restoration or resocialization (by facilitating the offender to make things right and to help the offender go on a better path/not commit crime again) can be an important reason to take part in VOM (Bolívar, 2013; Laxminarayan et al., 2015; Paul, 2015). However, individuals are likely to differ in their *a priori* attitudes toward programs (such as VOM) that help offenders resocialize (e.g., Maruna and King, 2009). We argue that:

These prior expectations (regarding the sincerity of the apology) and attitudes (toward resocialization programs) are likely to enhance the proposed visual attention–inference process through which the victims establish the perceived sincerity of the apology during a VOM encounter (H4).

### The Current Study

**FIGURE 1:**
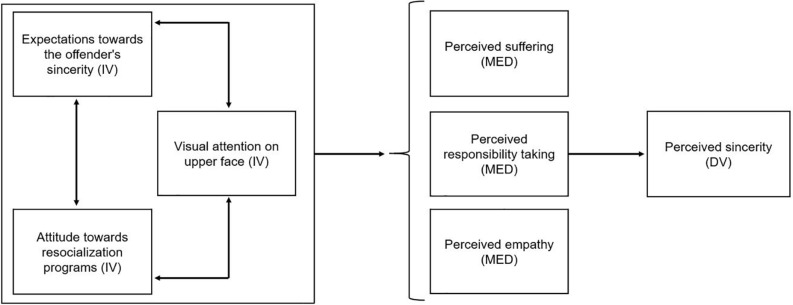
Proposed research model including the independent and mediating variables that together predict and explain the perceived sincerity of an offender’s apology in victim–offender mediation.

We propose that the victims will direct their gaze toward the offender’s eyes and eyebrows in order to detect emotions and inferences that are associated with a sincere, remorseful apology. The victim’s mental process to interpret such an apology might yet depend on expectations and attitudes the victims will hold before meeting the offender as well. The interplay of these distinct variables will be examined in this study. Figure 1 summarizes all dependent, mediating, and independent variables within the research model.

## Materials and Methods

### Participants

A total of 64 undergraduate students from the University of Twente in the Netherlands took part in this study. The stimulus material included a Dutch video from a previous study (Jonas-Van Dijk, 2016), in which an offender (i.e., a trained actor) offered a remorseful apology. We chose this apology because in this previous study this apology was validated to elicit substantial variations in perceived sincerity (see below in the paragraph on “The Apology of the Offender”). All instructions and the questionnaires were in English because the participants followed an English language program. Four participants indicated not to understand the Dutch video content sufficiently after participating and had to be excluded. Another participant was excluded due to the calibration inaccuracy of the eye tracking equipment. The first participant was also left out from further analysis because her trial served as a pilot test to finetune the study setup. Therefore, data from 58 participants (57%, *n* = 33 female; 43.1%, *n* = 25 male) between 17 and 30 years old (*M* = 21.26, *SD* = 2.99) were used for analysis. Most participants were Dutch (83%, *n* = 48); 17% (*n* = 10) were German. Most of them (80%, *n* = 48) were bachelor students of Behavioral Sciences (Psychology and Communication Science) and Engineering Technology (Creative Technology). The participants were approached through convenience sampling and could earn credit points or a small monetary compensation for their participation. About one-third of the participants (10 male, 10 female) indicated that they had been victimized at least once in their life, and the majority of the participants (67%; 19 male, 20 female) knew someone in their direct social network who had been victimized at least once in their lives. Several participants (12%; five male, two female) reported that they had committed a crime at least once in their life or knew a person in their direct social network that had committed a crime at least once (29%; 13 male, 4 female).

### Design

The participants were asked to take the role of a person who was victimized and – later on – attended a face-to-face mediation with the (fictitious) offender. A correlational design was adopted to test the associations between pre-measures (the attitude toward resocialization programs and the expectations toward the offender’s sincerity) eye movement data and post-measures, namely, the emotional inferences and the perceived sincerity of the apology. To determine the participants’ visual attention behavior on the offender (eye movement data), fixations and duration of eye movements were measured and analyzed in terms of their associations with the proposed variables. All procedures in this study were approved by the ethics committee of the Faculty of Behavioral, Management and Social Sciences of the University of Twente.

### Testing the Study Procedure

Before collecting data for the analyses, the experimental procedure was run and tested. For this purpose, the first participant was observed while doing the study and asked to share her thoughts and experiences about the procedure afterward. This pilot test showed that, for participants who wear glasses in daily life, an extra unit had to be put between the glasses’ lenses and the head unit of the eye tracker for measurement accuracy.

### Procedure and Materials

#### Overview of the Study

The participants were welcomed and they first read and signed an informed consent form that covered all aspects about the voluntary, confidential, and anonymous nature of the study. They were also informed that eye tracking technology would be used to trace and record their eye movement. The study was designed and conducted with the online software tool Qualtrics. After the participants gave their informed consent, they put on the head unit of the eye tracking device that was adjusted to the participant’s nose and head by the researcher. Subsequently, the glasses were calibrated to generate measurement accuracy by looking at a target mark (Ø 2.5 cm) that was placed at a distance of approximately 50 cm to the participant’s face. Then, measuring of the participant’s gaze behavior started and lasted through the whole trial in order for the participants to get used to wearing the glasses and not to disrupt their gaze behavior during the mediation scenario presented at a later stage of the study. The participants were instructed to imagine as good as possible being a victim in a violent burglary scenario that they were exposed to (through written information in Qualtrics) and were informed that the offender was arrested afterward. In the next phase, they received the information that they were approached by a mediator from an organization offering VOM in order to let them know that this offender would like to engage in mediated contact with them. Then, the basic principles of victim offender mediation (e.g., confidentiality, neutrality of the mediator, and voluntariness) were introduced to the participants, with the exception of presuming their agreement to participate in a face-to-face mediation with the offender. Also, the purpose of VOM was explained in order to provide an overview of how a natural face-to-face VOM could take place. Next, the participants’ expectations toward the offender’s sincerity and the participants’ attitude toward resocialization programs were assessed. They were then asked to contact the researcher who showed them the video clip in which the offender of the burglary scenario offered a remorseful apology to the victim. This stimulus material was not embedded in Qualtrics to facilitate showing of the video clip in a higher resolution. Subsequently, the participants indicated the degree of suffering, responsibility, and empathy that they perceived in the offender as well as the perceived degree of sincerity of the apology. At the end of the study, demographic data including gender, age, nationality, and current educational status were gathered. Also, own experiences with crime were recorded. Finally, text entry was given for personal remarks or questions concerning the study. Upon completion of the questionnaire, the gaze recording was stopped and the participants were debriefed.

#### Apparatus

Eye tracking data were collected simultaneously for both eyes with Tobii Pro Glasses 2, an eye tracking head unit device that sample eye movement every 4 ms (50 Hz) [product description of Tobii Pro Glasses 2 derived from https://www.tobiipro.com/siteassets/tobii-pro/product-descriptions/tobii-pro-glasses-2-product-description.pdf/?v=1.0.8 (2017)]. The head unit was connected with a recording unit that stored data on a 32-GB micro-SD card. A Dell tablet running Tobii Pro Glasses Controller analysis software was connected to the head unit and the main unit wirelessly for data acquisition. The informed consent, the self-report measures, and the stimulus material were presented on a 24” TFT LG Flatron W2442PE screen with a resolution of 1,920 × 1,080 pixels. The participants were seated in a distance of approximately 50 cm from the screen on a stable chair to reduce camera shaking through body movement during data collection. The data were mapped and corrected by comparing the motion pictures to a stable snapshot of the offender using Tobii Pro Analyzer running on Windows 10 software. Eye tracking metrics were inserted and analyzed in SPSS 24 in the dataset containing all self-report measures.

#### Victimization Scenario

The scenario of a violent burglary that was presented to participants in which they had to imagine being the victim was adopted and adjusted from Gromet and Darley (2011) [as also used by Jonas-Van Dijk (2016)].

On a Friday night, you go to an ATM machine to take out cash. You see nobody around you; it is a very calm night. You take your money from the machine into your wallet. Suddenly, you hear a noise and see someone approaching you. You feel a hard hit on your head and fall to the ground. The stranger is holding a gun in his hand, is pointing in your direction, and is shouting at you to give him your money. He grabs your wallet and runs away, leaving you lying on the sidewalk. No witnesses are around to give account to what happened. You are shocked and unable to chase the offender. The last thing you see is that he is running away. You feel a strong headache. You see blood on your hand after you intuitively touched your head. After several minutes, another person who comes to use the ATM finds you and calls 112. You are taken to the hospital; the next day, a police officer interviews you about the incident. Based on your description, the offender could be arrested and was convicted.

#### The Apology of the Offender

**FIGURE 2:**
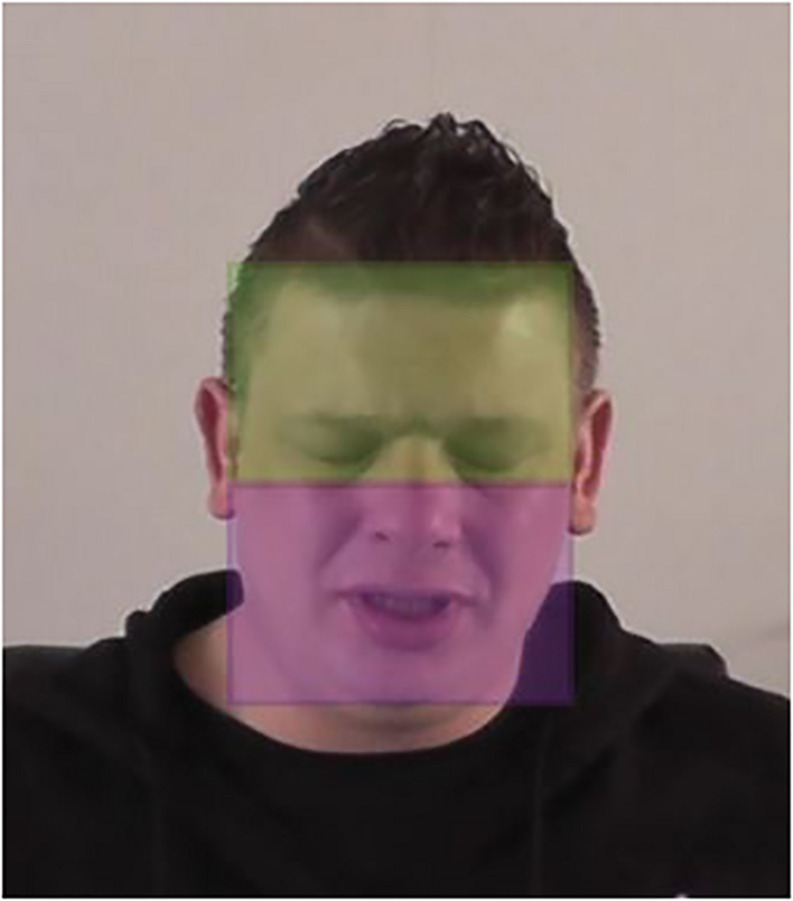
Areas of interest that represent the upper and the lower face, market on the snapshot of the stimulus material.

The participants were exposed to a video clip (length: 1 min 12 s) of an adult, White, Dutch-speaking actor who presented himself as the offender and offered his apology for his misdeed (for a snapshot, see Figure 2). He is sitting at a table in a neutral venue and directly addresses his apology to the viewer of the video through eye contact and use of the 2nd person. In this apology, the offender indicated his remorse for his offense, expressed that he was responsible for this, and acknowledged and expressed regret for the harm he had inflicted upon the victim. The content of the apology was developed in a previous research (Jonas-Van Dijk, 2016) and the actor was recruited from that researcher’s network; in that study, the participants (who also imagined being the victim of the above violent burglary; *n* = 126) indicated that they perceived this apology on average to be neither sincere nor insincere. That is, on a four-item scale of perceived sincerity [item example: “I doubt if the apology was sincere” (reverse coded); α = 0.92; answered on scales ranging from 1 (strongly disagree) to 7 (strongly agree)], the participants evaluated the apology as neutral in terms of sincerity (*M* = 3.80; *SD* = 1.41). These sincerity scores thus suggest that this apology evokes substantial variations in perceived sincerity among the recipients (in contrast to it being clearly insincere or highly sincere). The full text of the apology can be found in Appendix.

#### Independent Variables^2^

##### Attitude Toward Resocialization Programs

After the participants were instructed to put themselves in the role of a crime victim, seven items inquired on a five-point Likert scale (strongly disagree–strongly agree) on whether they generally prefer resocialization of offenders as a form of restorative justice policy over a retributive justice approach that focuses on the punitive treatment of convicted offenders. These items were inspired by Gromet and Darley (2011) to examine what people thought should be done to achieve justice in general. Five items were negatively formulated and therefore reverse-coded (e.g., *resocialization programs are a waste of time and money*). A higher score on this scale indicated that the participant had a more positive attitude toward restorative programs offered for offenders to reintegrate in the society (α = 0.73).

##### Expected sincerity of the offender

The victims’ initial expectation toward the offender’s sincerity before receiving the apology video was assessed with four items (one negatively formulated and thus reverse-coded) on a five-point Likert scale (strongly disagree–strongly agree) derived from the General Trust Scale (Yamagishi and Yamagishi, 1994). To fit the crime case scenario, the items were specified and directed toward the offender, for instance: *I think that the offender will tell a lie when he can benefit by doing so*. Reliability analysis revealed an acceptable alpha for this scale with α = 0.79.

##### Visual fixation and areas of interest

With Tobii Analyzer, a list of gaze data was obtained for each participant. Gaze behavior consists of (a) fixations that were defined as the amount of continuous time that was spent looking at a 20 × 20 pixels region and (b) eye movement that is necessary to inspect the whole visual scene in detail (Noton and Stark, 1971; Boraston and Blakemore, 2007). In the Tobii Software Analyzer, a minimal fixation time of 50 ms was set as a standard; this standard was also used in this study. The number of eye fixations on the whole visual area of the stimulus material was calculated for each participant, representing the locations and the sequences (saccades) of the eye fixations. Also, the duration of eye fixations (in seconds) was examined. In order to compare the fixation distributions, the offender’s face was categorized into look zones, each called AOI. In line with previous studies in the field of facial emotion recognition conducted by Wong et al. (2005) and Chaby et al. (2017), two AOIs were constructed. Both areas were set up to have the same size so that differences in gaze fixations between these areas did not occur because one area was larger. One was representing the upper face part (including the eyes and the eyebrows) and a second area covered the lower regions of the face (nose, mouth, and chin), as shown in Figure 2. The fixation numbers and durations were examined again related to these specific AOIs.

#### Mediator and Dependent Variables

##### Perceived suffering and responsibility taking

One scale including seven statements reflected on how the participants perceived the offender to be suffering from and to take responsibility for the consequences of his misdeed. Three items were reversed. They were adopted from the scale used in^1^, for instance: *The appearance of the offender indicates that he takes responsibility for the bad consequences of his deed*. Reliability was high for this scale with α = 0.83. An example for perceived suffering would be: *I doubt whether he is suffering emotionally from the effects of his actions* (α = 0.85). All items were measured with a five-point Likert scale (strongly disagree–strongly agree).

##### Perceived empathy

The participants were asked to indicate on a five-point Likert scale (strongly disagree–strongly agree) to what extent they perceive the offender to be expressing empathic feelings for them as a victim. Four statements were presented to measure perceived empathy, e.g., *The offender expresses empathy for the harm I suffer* (α = 0.73).

##### Perceived sincerity of the apology

Two scales assessed how sincere the offender’s apology was perceived to be. First, six statements were rated on a five-point Likert scale, ranging from strongly disagree to strongly agree; three statements were negatively formulated (and thus reverse-coded), for instance: *I have the feeling that he does not mean what he said to me*. After this, a second scale was presented, containing three questions regarding the offender’s sincerity, which was also rated on a five-point Likert scale (strongly disagree–strongly agree). These came from^1^ and were adjusted to the crime case scenario of this study. For example, we asked the participants: *Does the offender try to express different feelings than he actually has*? Reliability analysis revealed a high Cronbach’s alpha for both scales (α = 0.93 and 82, respectively). In a further analysis, both scales were taken together to measure the *perceived sincerity of the offender* (α = 0.95).

### Mapping Eye Fixations and Data Processing

We obtained a video showing the visual spectrum of each participant, including fixation points representing his or her gaze behavior. To compare and visualize the fixation distributions of all participants, we created a fixed snapshot that was most appropriate to represent an average frame of the position of the offender in the video. For each participant, every sequence with a value of 30 ms as minimum fixation duration was compared to the fixed snapshot to which a fixation point was added automatically by Tobii Analyzer. When the eyes rested on a visual area for at least 30 ms, the analysis tool added this as a fixation point to the output table. Every fixation point on the snapshot was compared to the corresponding video sequence. If the software did not map it accurately, the first author corrected it manually. The AOIs covering the two face regions of the offender were previously marked on the snapshot so that the fixation points could also be allocated to these regions by the analysis tool. A table with fixation data used in the analysis was obtained via the export function of Tobi Pro Analyzer so that eye tracking metrics could be inserted and analyzed in SPSS.

## Results

### Descriptives and Scale Construct Validity

Table 1 provides a correlation matrix of the main measurement scales containing their mean scores, standard deviations, and interscale correlations. The eye tracking data of fixation duration and number of fixations on the lower and the upper face area of the offender were listed separately and for the total snapshot including both AOIs and all remaining fixation points on the snapshot. The participants’ attitude toward resocialization programs had the highest mean score compared to the other scales, indicating that the participants generally had a positive opinion about a restorative treatment of offenders after a crime to foster his or her resocialization process. Both *expected sincerity* (prior to the apology video; *M* = 2.52, *SD* = 0.69) and *perceived sincerity* (after the video; *M* = 2.94, *SD* = 0.89) were above the midpoint of the scale. Notably, the perceived sincerity of the offender was significantly higher than the expected sincerity [paired-samples *t*-test, *t*(57) = -3.48, *p* < 0.005]. This means that the initial opinion about the offender’s sincerity improved after having seen the offender apologizing. Furthermore, the *perceived sincerity* correlated moderately to strongly with the emotional inferences; thus, as people detected higher feelings of *suffering*, *responsibility taking*, and *empathy*, they perceived the apology to be more sincere. Surprisingly, the expected sincerity did not significantly correlate with these emotional inferences, suggesting that these inferences are not associated with previous expectations. Against expectations, the eye tracking data did not correlate with the majority of all scales. However, a weakly positive, marginally significant correlation between the expected sincerity of the offender and the fixation duration on the upper face could be found. This suggests that higher expectations toward the offender’s sincerity seem to be related to a longer time spent looking at the upper area of his face. This finding thus seems to suggest that the participants consider the upper face area to be more informative during the reception of the apology to the degree that they expect the apology to be more sincere and therefore attracts more interest and demands more attention than the other visual areas.

**TABLE 1:** Interscale correlation matrix including the descriptives of all main variables for all participants (*N* = 58).

**Descriptives of main variables**	***M***	***SD***	**1**	**2**	**3**	**4**	**5**	**6**	**7**	**8**	**9**	**10**	**11**
1. Expected sincerity of offender	2.52	0.69	−										
2. Attitude resocialization programs	3.72	0.53	0.19	−									
3. Perceived responsibility	3.55	0.78	0.17	0.28*	−								
4. Perceived suffering	3.20	0.74	0.18	0.28*	0.70**	−							
5. Perceived empathy	3.39	0.72	0.14	0.31*	0.69**	0.65**	−						
6. Perceived sincerity of the apology	2.94	0.85	0.30*	0.40**	0.71**	0.80**	0.58**	−					
7. Fixation count lower face	42.62	35.95	–0.17	–0.03	–0.02	0.01	–0.02	–0.08	−				
8. Fixation count upper face	67.07	44.01	0.15	0.10	0.12	0.16	0.13	0.09	−0.45**	−			
9. Fixation count total snapshot	133.38	45.18	–0.05	0.06	0.10	0.23	0.12	0.01	0.49**	0.52**	−		
10. Fixation duration lower face	21.16	16.87	–0.21	–0.08	0.06	–0.02	0.03	–0.03	0.76**	−0.69**	0.00	−	
11. Fixation duration upper face	33.30	19.41	0.26	0.09	0.04	0.08	0.12	0.08	−0.76**	0.70**	–0.06	−0.84**	−
12. Fixation duration total snapshot	61.50	10.37	0.04	–0.03	0.23	0.22	0.31*	0.12	–0.61	–0.03	–0.08	0.22	0.29*

**^∗^p < 0.05; ^∗∗^p < 0.01; p = 0.051. The variables 1–6 were measured on a scale from 1 to 5. The variables 7–9 show the number (count) of eye fixations. Fixation duration (10–12) is the cumulated fixation duration indicated in seconds.**

### Eye Tracking Data and Hypotheses Testing

Before presenting the results related to the proposed relations between the inferences and the perceived sincerity in hypothesis 1, we first present the results related to the eye tracking data (hypotheses 2 and 3). We considered this logical as these data represent the visual attention of the participants on the offender’s face which preceded in time the inferences that the participants drew from the offender’s apology (to which hypothesis 1 relates).

### Fixation distributions on all parts of the visual area including AOIs

**FIGURE 3:**
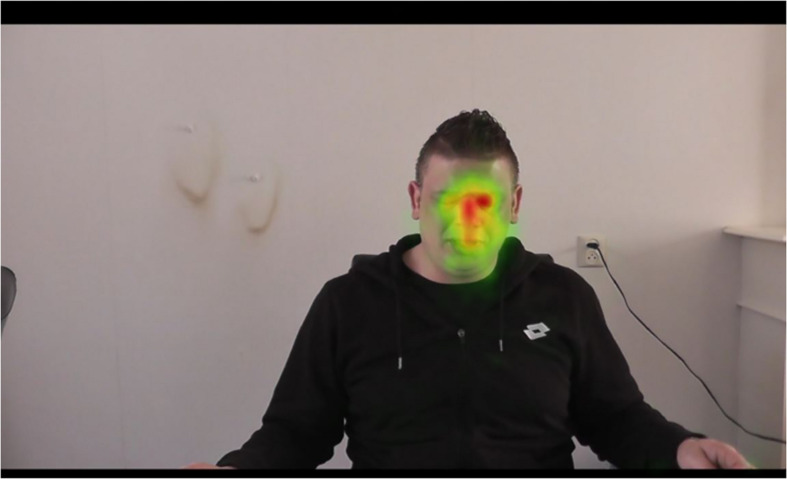
Heat map of the participants’ visual attention to the offender who apologizes for his misdeed.

**FIGURE 4:**
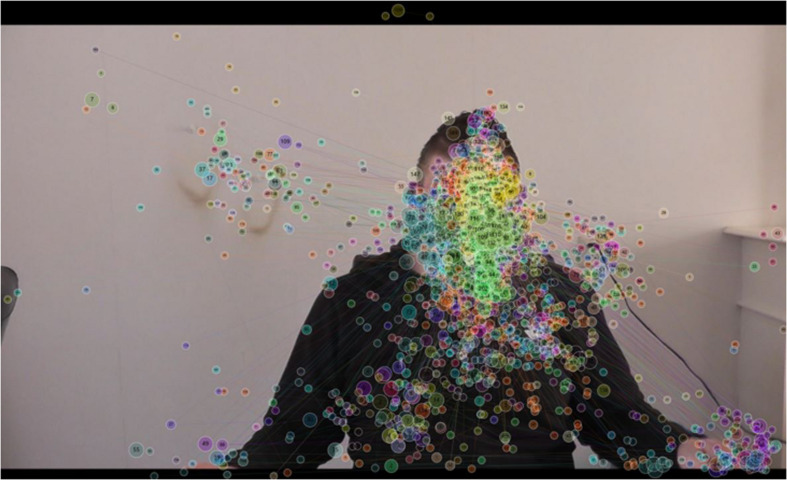
Gaze plot indicating the location, the gaze sequence, and the time spent looking at the stimulus for every single participant (colored).

Figures 3, 4 provide a visualization of the (a) fixation duration and (b) fixation count that was acquired from the 58 participants, represented on the snapshot that was used to map eye tracking metrics. Fixations that were outside the computer screen were left out for further analysis as they did not relate to the visual area of the apology–mediation scenario. Figure 3 illustrates the distribution of the visual attention (gaze behavior) of all participants, summarized in a heat map. It provides a heuristic overview of all data points; the order of fixations, the individual scan paths, and the minor fixations are not visualized. The center of the heat map, indicated by warm colors such as red and orange, shows that the fixation focus of all participants predominantly lies on the left eye of the offender and the space between both eyes, including the upper part of the nose. Colored in green, the visual areas around the fixation focus, mainly consisting of the right eye, the forehead, and the mouth, also gained the attention of the participants but to a smaller degree.

Figure 4 provides a visualization of all gaze data in detail. This gaze plot was created to show the viewing pattern including location, gaze sequence, and time of attention distribution for every participant apart – each color represents a different participant. Each circle in Figure 4 represents the time a participant was looking at a fixation point (fixation duration). To the degree that a participant looked longer at a fixation point, the larger was the diameter of (and thus) the circle. A divergent distribution of fixation points on all areas of the stimulus material can be seen; notably, the density of fixation points on the offender’s face is higher than on the remaining areas.

Subsequently, we compared the participants’ number of eye fixations on the predefined AOI of the lower and the upper face parts. As expected, a paired-samples *t*-test showed that there was a significant difference between the average number of fixations on the lower face part (*M* = 42.62, *SD* = 35.95) and the fixation count on the upper face part [*M* = 67.07, *SD* = 44.01, *t*(57) = −2.73 and *p* = 0.037], with the latter gaining higher visual attention. Additionally, a comparison of the duration of visits and fixations of all participants on the lower and the upper face parts of the offender revealed that they also spend significantly more time (in seconds) to fixate on the upper face of the offender (*M* = 33.30, *SD* = 19.41) than on the lower face part [*M* = 20.17, *SD* = 11.39, *t*(57) = -2.66, *p* = 0.031]. As expected, these divisions thus indicate that visual attention was more strongly directed to the upper face part, including eyes and eyebrows, than it was to the lower face part, including the mouth and the chin, of the offender. These findings together offer support for our hypothesis 2 that during the observation of the offender who gives his apology, the victims’ attention is focused more on the upper face area than on the lower face area of the offender. Of course, during any face-to-face conversation, people may be inclined to spend most time on their conversation partner’s upper face area as a way to express active listening (Rastogi, 2008; Keyser, 2013). The important question therefore is to what extent the independent, mediator (inference), and dependent variables in this study relate to the eye gazing behavior of the participants.

In the next paragraphs, the results concerning the interplay between previous expectations and *a priori* attitudes toward resocialization, gaze behavior, and perceived emotional inferences and how these predict the perception of the offender’s sincerity are examined. In line with the temporal order that we proposed in the research model in Figure 1, a hierarchical regression analysis was conducted to test predictions about the perceived sincerity, which served as a dependent variable in each model. In total, three models were included in the analysis. Model 1 contained the attitude toward resocialization programs and the expectations of the offender’s sincerity. The second model added the victims’ eye tracking metrics. Subsequently, inferences about the perceived suffering, responsibility taking, and empathy the offender expressed toward the victim were added to the analysis in the third model. Table 2 summarizes all variables within each model, including *B*’s, standard deviations, and *p*-values.

**TABLE 2:** Regression model including B, SE_B_, and *p* for every predictor.

	**Model 1**			**Model 2**			**Model 3**		
	***B***	**SEB**	***p***	***B***	**SEB**	***p***	***B***	**SEB**	***p***
Perceived attitude resocialization	**0.58**	**0.20**	**0.005**	**0.58**	**0.20**	**0.006**	**0.26**	**0.13**	**0.046**
Expected sincerity toward offender	**0.30**	**0.16**	0.069	0.30	0.16	0.069	0.15	0.10	0.121
AOI fixation count upper face				0.00	0.00	0.498	0.00	0.00	0.500
AOI fixation count lower face				0.00	0.01	0.371	0.00	0.00	0.241
AOI fixation duration upper face				0.00	0.01	0.849	−0.01	0.01	0.527
AOI fixation duration lower face				0.01	0.01	0.353	0.00	0.01	0.895
Perceived suffering							**0.66**	**0.13**	**0.000**
Perceived responsibility taking							**0.31**	**0.13**	**0.020**
Perceived empathy							−0.05	0.13	0.737

**For every model, the perceived sincerity of the offender was the dependent variable.* Bolded values are significant values.*

#### Attitudes Toward Resocialization Programs Predict the Perceived Sincerity of the Apology

Model 1 was statistically significant with *R*^2^ = 0.21, *F*(2, 55) = 7.36, and *p* = 0.001. The analyses of the regression coefficients are displayed in Table 2. As expected, the general attitude toward resocialization programs positively predicted the perceived sincerity of the apology (*B* = 0.58, SE*_B_* = 0.20, *p* = 0.006). In addition, expected sincerity tended to predict perceived sincerity as well, although not significantly (*B* = 0.30, SE*_B_* = 0.16, *p* = 0.069). Model 1 thus indicates that victims who have a more positive attitude toward resocialization programs and have higher expectations regarding the sincerity of the offender also (tend to) perceive his apology to be more sincere after having received and watched the apology video.

#### Gaze Behavior Does Not Directly Predict Perceived Sincerity

Model 2 added eye tracking metrics, including fixation count and durations for both AOI of the lower and the upper face. Against expectations, adding these metrics did not significantly improve the model [*R*^2^_change_ = 0.02, *F*(4, 51) = 0.33, *p* = 0.858]. As a whole, model 2 was also not statistically significant with *R*^2^ = 0.30, *F*(4, 48) = 2.25, and *p* = 0.84. These findings indicate that, unexpectedly, the eye tracking metrics of the victim cannot be regarded as direct predictors for the evaluation of the sincerity of the offender’s apology. Table 2 shows that none of the four predictors that were added to the model were significant predictors of perceived sincerity. The previous significant predictors from model 1 exerted the same influence in model 2 (see Table 2). Hypothesis 3, which proposed that the fixations on the upper face part of the offender predict the inferences of suffering, responsibility taking, and empathy and in turn the perceived sincerity of the apology, could therefore not be supported based on our findings. That is, the descriptive analyses (correlations) revealed that the fixations on the upper face part were unrelated to these three inferences, and these regression analyses indicate that these fixations did not predict perceived sincerity as well.

#### The Importance of Perceived Suffering and Responsibility Taking (but Not Empathy) in Predicting Perceived Sincerity

Model 3 added perceptions about the apology of the offender, including inferences of responsibility taking, perceived suffering, and perceived empathy. Adding these inferences resulted in a significant improvement of the model [*R*^2^_change_ = 0.51, *F*(3, 48) = 30.84, *p* < 0.01]. The predictors in model 3 together explained a significant amount of variance in perceived sincerity [*R*^2^ = 0.76, *F*(4, 44) = 10.71, *p* < 0.01]. In line with our expectations, perceived suffering (*B* = 0.66, SE_B_ = 0.13, *p* < 0.05) and perceived responsibility taking (*B* = 0.31, SE_B_ = 0.13, *p* < 0.05) positively and significantly predicted the perceived sincerity of the apology. This means that to the extent that the participants inferred that the offender suffered and took responsibility, the more they perceived the apology to be sincere. Unexpectedly, however, perceived empathy did not predict the perceived sincerity of the offender to a statistically significant degree in this model (*B* = -0.05, SE = 0.13, *p* = 0.74). Therefore, hypothesis 1 is partly confirmed. Expected sincerity, that was a significant predictor in the first model, was now no longer significant (*B* = 0.15, SE_B_ = 0.10, *p* = 0.12). The attitude toward resocialization programs remained significant, but its effect on perceived sincerity was weakened compared to model 1.

#### Do Perceived Suffering and Responsibility Taking Mediate the Relation Between Expected Sincerity and Attitude Toward Resocialization Programs on Perceived Sincerity?

The above analyses suggest that perceived suffering and responsibility taking (but not empathy) mediate the effect of expected sincerity and attitude toward resocialization programs on perceive sincerity, which is congruent with our proposed research model. Therefore, we conducted two separate mediation analyses. First, to test the effect of expected sincerity on the perceived suffering and responsibility taking of the offender and the perceived sincerity, mediation analysis was conducted with the expected sincerity as predictor, the perceived suffering and responsibility taking as mediators, and the perceived sincerity as criterion variable. We left out the eye metrics as these did not relate directly to expected or perceived sincerity in this analysis – as a result, the outcomes of this analysis are different compared to comparable outcomes in Table 2.

In step 1 of the mediation model, the regression of expected sincerity on perceived sincerity was significant [*B* = 0.36, *t*(1, 56) = 2.31, *p* = 0.025], indicating a total effect of expected sincerity on the perceived sincerity. Step 2 showed that the regressions of expected sincerity on the mediators perceived suffering [*B* = 0.20, *t*(1, 56) = 1.40, *p* = 0.167] and responsibility taking [*B* = 0.19, *t*(1, 56) = 1.29, *p* = 0.202] were not significant, indicating no indirect effects of expected sincerity on the mediators within the model. In step 3 of the mediation process, the effects of the mediators on perceived sincerity were significant with *B* = 0.65, *t*(3, 54) = 5.37, and *p* < 0.001 for perceived suffering and *B* = 0.32, *t*(3, 54) = 2.85, and *p* = 0.006 for perceived responsibility taking. Step 4 of the analysis returned that the direct effect of expected sincerity on perceived sincerity was not significant, with *B* = 0.17, *t*(3, 54) = 1.85, and *p* = 0.070. Taken together, these findings suggest that there is no mediation effect between expected sincerity, perceived suffering, and responsibility taking and perceived sincerity.

**FIGURE 5:**
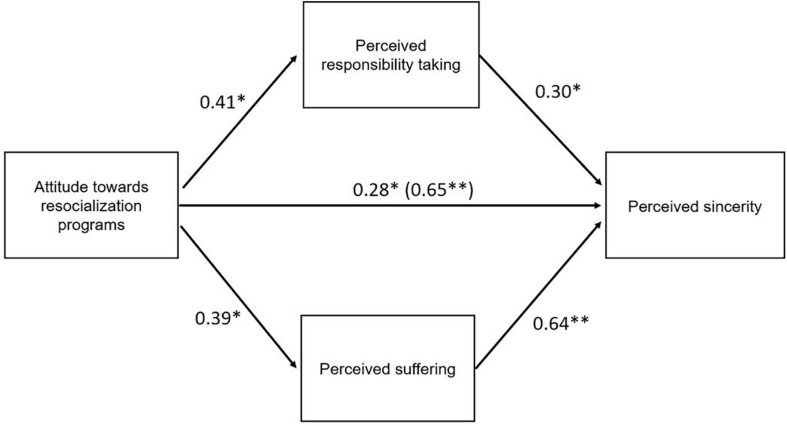
Perceived suffering and responsibility taking partially mediate the relationship between the attitude toward resocialization programs and the perceived sincerity of the offender (*p < 0.05; **p < 0.001) (The *B*-value of the total effect of attitude toward resocialization programs on perceived sincerity is shown in brackets).

Second, to test the effect of the attitude toward resocialization programs on the perceived suffering and responsibility taking of the offender and the perceived sincerity, mediation analyses were conducted with attitude toward resocialization programs as predictor, the perceived suffering and responsibility taking as mediators, and the perceived sincerity as criterion variable. In step 1 of the mediation model, the regression of attitude toward justice systems on perceived sincerity was significant [*B* = 0.65, *t*(1, 56) = 3.28, *p* = 0.002], indicating a total effect of attitude toward justice systems on the perceived sincerity. Step 2 showed significant indirect effects of attitude toward justice systems on the mediators perceived suffering [*B* = 0.39, *t*(1, 56) = 2.15, *p* = 0.036] and responsibility taking [*B* = 0.41, *t*(1, 56) = 2.15, *p* = 0.036]. In step 3 of the mediation process, the effects of the mediators on perceived sincerity were significant with *B* = 0.64, *t*(3, 54) = 5.33, and *p* < 0.001 for perceived suffering and *B* = 0.30, *t*(3, 54) = 2.71, and *p* = 0.009 for perceived responsibility taking within the mediation model. Within the model, attitude toward resocialization programs is a significant positive predictor as well as perceived suffering and responsibility taking (as can be seen in Table 3). Figure 5 shows the effects of the predictor on the mediators and the dependent variable. Step 4 of the analysis returned that the direct effect of attitude toward justice systems on perceived sincerity was meaningfully reduced by adding the mediators in the model, with *B* = 0.28, *t*(3, 54) = 2.22, and *p* = 0.03. Taken together, these findings suggest that perceived suffering and responsibility taking partially explain why the attitude toward resocialization programs is a positive predictor of perceived sincerity.

**TABLE 3:** Regression coefficients and confidence interval for attitude toward resocialization programs, suffering, and responsibility taking, with perceived sincerity as the dependent variable.

					**95% CI**	
	**B**	**SE_B_**	***t***	***p***	**Minimum**	**Maximum**
Attitude toward resocialization programs	0.28	0.12	2.22	0.03	0.03	0.53
Perceived responsibility taking	0.30	0.11	2.71	0.01	0.08	0.53
Perceived suffering of offender	0.64	0.12	5.33	0.00	0.40	0.87

#### Attention on the Offender’s Upper Face: The Interaction Between Attitude Toward Resocialization Programs, Gaze Behavior, and Inferences of Responsibility Taking

As described above, so far, the gaze behavior toward the upper face area of the offender of the participants was not related directly to their inferences and perceived sincerity. As we indicated in our research model, we did not have any predictions regarding the specific interplay between the *a priori* variables expected sincerity, attitude toward resocialization programs, and participants’ gaze behavior and on their combined influence on the inferences of suffering and responsibility taking. We generally expected that expected sincerity and attitude toward resocialization programs would enhance the proposed visual attention–inference process through which victims establish the perceived sincerity (hypothesis 4), but as there are no robust, direct relations observed between sincerity/attitude and gaze behavior and between gaze behavior and inferences, this hypothesis could not be supported.

We therefore conducted additional exploratory analyses to detect whether the gaze data concerning the upper face area of the offender (fixation count and duration) perhaps moderated the relations between expected sincerity and attitude toward resocialization programs on the one hand and the inferences of suffering and responsibility taking on the other. One of these four relations indeed appeared to be moderated significantly by the participants’ gaze behavior. That is, the moderation analysis with attitude toward resocialization programs as predictor, the fixation duration on the upper face as continuous predictor, and their interaction effect as predictor with perceived responsibility taking as criterion variable showed a significant interaction effect (see Table 4).

**TABLE 4:** Regression coefficients and confidence interval for the predictor’s attitude toward resocialization programs, fixation duration on the upper face, and their interaction, with perceived responsibility taking as the dependent variable.

					**95% CI**	
	***B***	**SE_B_**	***t***	***P***	**Minimum**	**Maximum**
Attitude toward resocialization programs	−0.33	0.39	−0.85	0.40	−1.12	0.45
Fixation duration on upper face area	−0.074	0.04	−2.14	0.04	−0.14	−0.005
Interaction fixations and attitude	0.02	0.01	2.18	0.03	0.00	0.04

**FIGURE 6:**
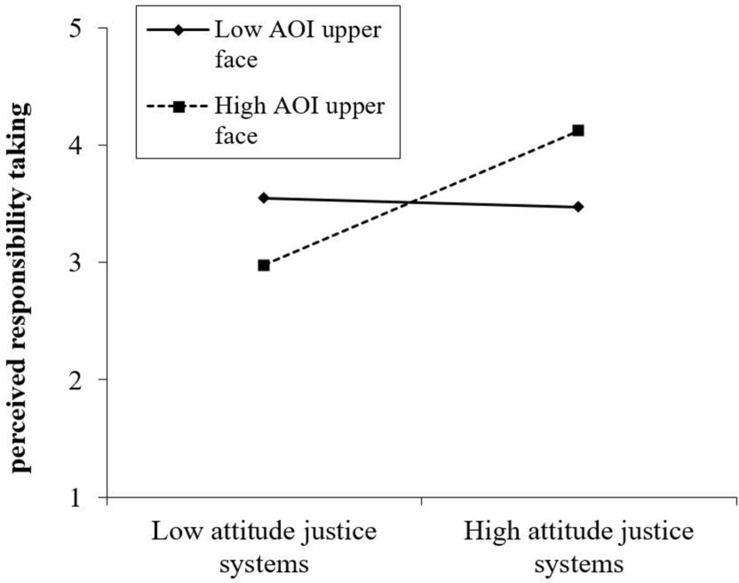
Two-way interaction between attitude toward resocialization programs and fixation duration on the upper face on perceived responsibility taking. AOI, area of interest.

In Figure 6, we have plotted this interaction effect at low (1 SD below the mean) and high (1 SD above the mean) levels of attitude toward resocialization programs and fixation duration on the upper face area of the offender. Most interestingly, the pattern indicates again (see Table 1) that attitude toward resocialization programs is associated positively with inferred responsibility taking, but only when the participants’ gaze was fixated relatively long on the upper face area of the offender. When the participants spent relatively little time fixating their gaze on the upper face area, this positive association was attenuated. Put differently, the pattern indicates that for those who have a relatively positive attitude toward resocialization programs, fixating the gaze longer (versus shorter) on the upper face area of the offender predicted an increase in perceived responsibility taking. In contrast, for those with relatively negative attitudes toward resocialization programs, fixating the gaze longer (versus shorter) on the upper face area had the opposite impact: for them, it decreased perceived responsibility taking. These results thus suggest that looking at the upper face area of the offender increases responsibility taking for those who favor resocialization programs, whereas looking at the same area of the offender’s face decreases responsibility taking for those who are more skeptical about resocialization.

#### Summary of the Results

**FIGURE 7:**
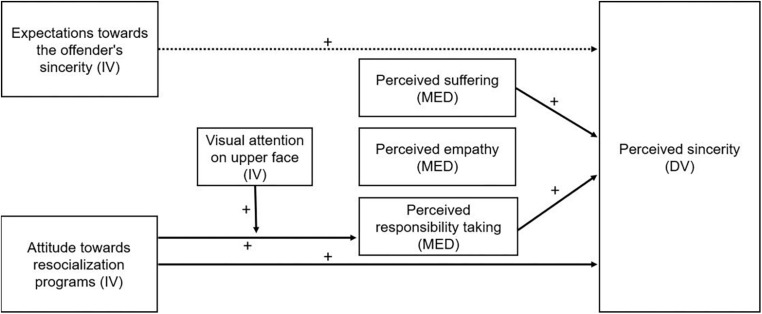
Summary of the main findings with regard on the proposed research model, indicated with solid arrows.

Figure 7 summarizes the most inclusive results obtained from the analyses with regard expectations ascribed in the initial research model.

## General Discussion

To our knowledge, this study was the first to use eye tracking to examine how victims might distribute their attention in terms of eye movement and gaze fixation durations while watching an offender giving an apologetic statement about his wrongdoing in the context of VOM. By conceptualizing the association between the victims’ *a priori* attitudes and expectations about the offender, where victims look at during a fictitious face-to-face mediation, and how they interpret the offender’s apology in terms of responsibility taking, suffering, and perceived sincerity, we sought to address the lack of knowledge about the way the victims might come to conclusions about the offender’s apology during mediation (but see Choi and Severson, 2009). Receiving a sincere apology from the offender plays a central role in the mediation process and is consistently regarded as one of the major mechanisms through which VOM has positive outcomes for the victims (e.g., Shapland et al., 2007; Dhami, 2012), yet, in practice, this often remains an unfulfilled desire when the victims do not perceive the offender’s apology as sincere, leading to detrimental consequences such as less satisfaction with the mediation and less willingness to forgive the offender. A substantial body of research explains how mediation may elicit positive effects for those who participate (e.g., Umbreit et al., 2005; Strang et al., 2006; Jacobsson et al., 2012; Van Camp and Wemmers, 2013; Van Camp, 2016; Abrams et al., 2017; Hansen and Umbreit, 2018). Surprisingly, as also asserted by several scholars, the emotional dynamics within the restorative process remain largely unclear (Choi et al., 2010). Within the current study, we assumed that distinct (1) previous, (2) visual, and (3) emotion-inferential factors might promote or hinder the positive reception of an offender’s remorseful apology among victims given by an offender in a face-to-face mediation scenario. Understanding these processes will provide in-depth knowledge of *how* a direct, face-to-face conversation might attenuate the victim’s perceptions and will help to facilitate more promising mediation outcomes for both parties.

### Main Findings

Our results show that the victims’ attention was primarily distributed among the offender’s upper face region, i.e., around the eyes and the eyebrows (e.g., Chaby et al., 2017). Eye fixations were more often and longer directed toward this region in comparison to all other visual areas, including the offender’s lower face part. Against expectations, however, our analyses showed that these parameters could not be linked directly to apology-related inferences (but indirectly instead; see below). A possible explanation for these unexpected findings might be straightforward: Human interaction in communication often comprises eye contact as a means of active listening (Rastogi, 2008; Keyser, 2013). The participants might have (unconsciously) applied their active listening skills to manage the tasks properly. For instance, before the stimulus material was presented, they were instructed to judge the apology afterward. Keeping this in mind, the participants might have been encouraged to remain in eye contact regardless of the verbal and the non-verbal information of the offender, expressing that they were carefully watching the apology and also knowing that their gaze data were recorded.

We further examined how distinct *a priori* and perceptual factors particularly determine the assessment of the apology. This study demonstrates that the general standpoint the participants had toward programs applied to facilitate an offender’s resocialization (VOM being a typical example of such a program) substantially accounted for the perceived sincerity. In other words, for participants who were more in favor of these programs (i.e., believing that everyone deserves a second chance in life), the offender appeared to be more sincere than for those who had a less positive opinion about such programs attempting the societal reintegration of offenders. Similarly, expectations toward the sincerity of the offender contributed to the perception of the sincerity as well, yet to a less significant degree. Building on previous research, this paper provides support that perceived suffering and responsibility taking of the offender are associated with a sincere apology: the previous findings of^1^ could be replicated with this study. As hypothesized, the more the offender is perceived to suffer from his misdeed and to take responsibility for what he did, the more his apology is perceived as sincere. Furthermore, our initial expectations suggested that empathy also affects how the victim perceives the offender who offers his or her apology. Surprisingly, no evidence was found for this in the current study, contrasting to what the earlier research proposes (Choi and Severson, 2009; Roschk and Kaiser, 2013). For this, the understanding of each concept might provide a comprehensible explanation: By (emotionally) suffering, one admits that he or she is seriously affected by the misdeed as well; in the same sense, taking responsibility indicates accountability for the harmful consequences a misdeed could have caused. Comparing these findings, self-critical inferences such as suffering and responsibility taking might have a stronger potential to embody genuine commitment than expressing empathy which may have less discriminating potential to express self-blaming perceptions. The offender, responsible for the negative consequences of his or her misdeed, may be perceived as more trustworthy when actively expressing accountability instead of simply showing empathy for the victim’s losses.

As a final explorative but important finding, this study suggests how gaze behavior may regulate the relationships between *a priori* attitudes toward resocialization programs and perceptual cues in mediation: The fixation duration on the offender’s upper face moderates the way in which the general attitude toward resocialization programs predicts the degree of responsibility taking inferred from the offender. This effect changes, depending on how much time people spend looking at the upper face region. If their attention was focused on this area for a relatively short time, the attitude toward resocialization programs did not predict the level of responsibility taking inferred, yet if their attention was directed to the offender’s upper face region for a longer period of time, the amount of perceived responsibility taking *increases* among those who have a positive attitude toward resocialization, whereas it *decreases* for those who are more against resocialization.

Importantly, this finding may explain for whom face-to-face mediation has, due to its capacity to provide non-verbal cues, a higher potential to bring about a beneficial mediation process (as responsibility taking predicted positively also perceived sincerity) and for whom it may not. In the context of the existing literature, this finding further extends what is known about the (positive) reception of a remorseful apology as little is known about the processes that cause positive outcomes for both victim and offender (e.g., Rypi, 2017). By this, it becomes clear what the advantages and risks of participation in a direct, face-to-face mediation may be and for whom: those who are open to resocialization of the offender may use the (non-)verbal information of the offender to conclude that s/he is genuine, whereas those who disagree with resocialization may see in the same (non-)verbal information more signs and proof of the insincerity of the offender, which can have, in turn, a negative impact on the victim as well as the offender during the mediation process (Bolívar, 2013; Hansen and Umbreit, 2018).

### Limitations of the Study

The current study is not without limitations. First and foremost, these are related to the setup of this study, which took place in an artificial experimental environment. We presented an apology given by a confederate and were interested in the participants’ gaze pattern. Data were gathered from college students who were instructed to imagine that they were victimized and that they were approached to participate in VOM by a mediator. In a natural VOM setting, it would not have been possible to do this in a standardized way except if all victims would have experienced the same crime and commonly received the offender’s apology. However, the participants’ perceptions may differ from the behavior and the negative emotions and feelings the target population (victims) might experience after a crime and during mediation (although a fair share of the participants indicated that they had been a victim of crime in their lives; 34.5%) (see Shnabel and Nadler, 2008, 2015). This might also affect the gaze behavior and the attention fixations on the offender (Schulze et al., 2013). Despite these differences, however, individuals of the target population can be argued to use similar visual and mental strategies to evaluate the apology as the participants in the current sample. This seems to be underscored by the fact that we did not observe any correlations between pre-measures of fear and anger toward the offender and any of the gaze data measures in this study. Put differently, there were no differences between the participants reporting very high levels of fear and/or anger toward the offender (resembling the experience of actual victims of harmful crimes) and those reporting lower levels in terms of their gaze behavior. Also, the time elapsed since the offense importantly has to be considered to generate more elaborate findings towards the measure of fear and anger (Zebel, 2012). In line with this consideration, a second limitation needs to be examined. We simplified the mediation procedure by leaving out several steps that usually take place in the mediation process in order to avoid this study being unnecessarily complicated to the participants. To make this study as generalizable as possible, we presented a violent burglary scenario which is (next to vandalism, minor assault, and theft) one of the four most common offenses that preceded VOM (Hansen and Umbreit, 2018). The scenario presented in this study might be regarded as an example of how a VOM could look like but does not claim entire validity for all possible VOM scenarios. Arguably, the type of crime will have a major influence on a victim’s attitude toward an offender. Our study may therefore not be able to examine the potential of VOM fully or in its entirety given the fact that each mediation has its unique characteristics and circumstances that preceded the encounter between a victim and an offender. Application of technology in natural VOM requires a careful consideration of the specific circumstances in which the victim and the offender face each other. Future investigations, if conducted in a natural VOM, should examine the victims’ gaze behavior more specifically with regard the different crime contexts, circumstances, and characteristics of the participants.

Accordingly, not only crime characteristics but also individual differences such as age, gender, or cultural background might influence the victim’s behavior and, consequently, his or her fixation distribution on the offender’s face. Literature reveals cultural differences in the way humans perceive emotions in others’ faces and how they direct their attention to recognize others’ mental states and to maintain social interaction (e.g., Blais et al., 2008; Akechi et al., 2013; Uono and Hietanen, 2015). Our study has been conducted within a Western, individualistic cultural setting. Our findings are likely to be less applicable to Eastern collectivistic cultures where communication tends to be more evasive, affecting both eye gaze display and interpretation (Akechi et al., 2013). There is also evidence that people from collectivistic cultures perceive and process facial stimuli in a more holistic way (Kelly et al., 2010). Uono and Hietanen (2015) also state that maintaining eye contact might evoke contrasting associations among cultures: Western Europeans positively evaluate eye contact in a social interaction; in contrast, in East Asian cultures, this is often perceived as a sign of disrespect (Uono and Hietanen, 2015). However, given the emotional dynamics within VOM, it is not entirely clear whether and how these culture-specific principles might also apply to differences in the victims’ gaze distribution in the context of VOM. As mentioned in the second limitation, VOM needs to be regarded as an exceptional setting that might be far from common, daily-life social interactions at times. For instance, the question arises if a victim deliberately would maintain eye contact toward the offender to express disrespect. More research is needed to test if the mental processes that take place during VOM converge with culture-specific values. Third, some participants indicated that the apology seemed to be scripted and that their evaluation of the apology was negatively influenced by this. However, this is not necessarily at odds with actual mediation practice, in which victims sometimes also indicate that the apology expressed by the offender did not seem very authentic (Choi and Severson, 2009). Additionally, the offender was not present *in vivo* as is the case in the majority of VOM cases. Awareness of the offender’s physical proximity can cause a major concern and stressful feelings for many victims when attending mediation (Shapland et al., 2008). For this (and other) reasons, many VOM programs around the world also offer other indirect forms of VOM, such as letter exchange or sending and receiving video messages (Larsen, 2014; Perspectief Herstelbemiddeling, 2019). In that sense, the video method used in this study has ecological validity. Fourth, we assumed the victims’ willingness to engage in VOM as the victims were solely informed about the possibility to have mediated contact with the offender. Notably, VOM follows the principle that it is entered into on a voluntary basis (Umbreit and Armour, 2010; Ponce-lópez et al., 2015; Hansen and Umbreit, 2018). We did not control whether a participant would have been willing to take part in mediation or not. By this, under-average datapoints of initial expectations toward the offender’s sincerity might be explained. Low expectations toward the offender’s sincerity are found to be a predictor why victims decline engagement in VOM (e.g., Wemmers and Canuto, 2002). Importantly, Jonas-Van Dijk (2016) compared the victims who engaged voluntarily or involuntarily in VOM. Those who did not voluntarily take part in VOM perceived the apology to be less sincere than those who took part voluntarily. Importantly, comparing these two groups, there was no difference between the relation of perceived suffering and responsibility taking and perceived sincerity (Jonas-Van Dijk, 2016).

Finally, wearing glasses during the study might feel unfamiliar for those participants who are not used to wearing glasses in daily life. Given the fast development of eye tracking technology, we might consider different solutions (for instance, a stable eye tracking device that is put in front of the participant) to track the participants’ gaze behavior in a follow-up research.

## Conclusion and Future Directions

Modern eye tracking techniques rapidly developed over recent years and provide a more accurate and valid detection of gaze behavior than in the years before (Funke et al., 2016). The current study exemplifies how technology and eye tracking in particular might be applied in VOM in order to understand how a victim might process the non-verbal behavior of the offender and how this might influence the victim’s conclusion of his or her sincerity. With this study, we also propose that the application of novel technology, for instance, the development of digital communication forms, will substantially enhance VOM.

Not only for VOM but also within related fields this technology might be a beneficial means – perhaps in combination with other non-invasive measurements – to provide valuable data in a very accurate and valid way. For instance, research has shown that attitudes (toward sexual violence) are associated with offender perceptions of whether their offending behavior caused their victim harm, victim empathy, and victim selections (Debowska et al., 2018; Debowska et al., 2019). Similarly, these attitudes are also predictive of juror beliefs in defendant and complainant stories at trial, credibility and sincerity assessments, and overall verdict decisions (Willmott et al., 2018). To further investigate these judgments and decisions, eye tracking technology might provide a highly effective means.

For future research, we conclude that eye tracking technology offers substantial potential to gain insight into cognitive and inferential processes that have not been studied before. This paper provides an exploratory approach to apply eye tracking in a simulated victim offender mediation scenario. Considering the fact that VOM programs are applied in a wide range of contexts, more differentiated research is needed toward new directions: In particular, a more process-related research approach will provide more in-depth knowledge about the (un)conscious, emotional processes involved in VOM that might be linked to the beneficial outcomes VOM can produce for victims as well as offenders (Shapland et al., 2007, 2008). This study underlines the importance of such an in-depth approach: receiving and looking at the non-verbal behavior in the upper face of the offender during his apology predicted quite diverging inferences of responsibility taking on the part of the victims, depending on whether they favored or dislike offender resocialization. In turn, these differences in perceived responsibility taking predicted concurrent evaluations of the sincerity of the apology – which is one of the major outcomes of the VOM process for victims (Laxminarayan et al., 2015). These findings suggest that it is important to take into account the victims’ *a priori* orientations toward resocialization in the mediation process as it influences what impact it has for them to look the offender in the eye when making an apology.

## Data Availability Statement

The datasets generated for this study are available on request to the corresponding author.

## Ethics Statement

The studies involving human participants were reviewed and approved by Ethics Committee of the Faculty of Behavioral Sciences, University of Twente, Enschede. The patients/participants provided their written informed consent to participate in this study. Written informed consent was obtained from the individual(s) for the publication of any potentially identifiable images or data included in this article.

## Author Contributions

EG, SZ, and FB designed the experimental setup. FB collected the data, while SZ and FB analyzed the data. FB wrote the manuscript, while EG and SZ edited the manuscript.

## Conflict of Interest

The authors declare that the research was conducted in the absence of any commercial or financial relationships that could be construed as a potential conflict of interest.

## Footnotes

1 Zebel, S., Giner-Sorolla, R., and Kamau, C. (2020). Suffering and responsibility-taking inferences explain how victim group members evaluate wrongdoers’ expressions of negative feelings (unpublished manuscript).

2 A note on additional measurements. In order to allocate this study in the context of contemporary VOM- research examining the beneficial (practical) effects of VOM, such as healing the victim’s emotional harm or reduce the offender’s risk to reoffend, we included additional measures in the research instrument. For the sake of conciseness of this article, these measurements were not included in the final version of the proposed research model. Additional measurements are listed in the Supplementary Material.

## Appendix

### The Content of the Apology (English Translation Below)

Hallo, Allereerst wil ik u bedanken dat u contact met mij hebt willen hebben. Daar heb ik er erg veel respect voor. Ik kan me voorstellen dat het voor u erg lastig moet zijn om mij op welke manier dan ook onder ogen te komen. Voor mij is het ook moeilijk.

Ik kan me ook voorstellen dat u de afgelopen periode erg bang hebt moeten zijn door wat ik gedaan heb. Ik wil dan ook graag zeggen dat het me enorm spijt. Ik weet nu hoe fout ik zat en dat het verschrikkelijk is wat ik u heb aangedaan. U heeft alle recht om boos op mij te zijn, want dit had u niet mogen overkomen. Het had niemand mogen overkomen. Ik zal mijn daden nooit goed kunnen praten en dat is ook niet wat ik wil. Ik wil alleen heel graag mijn excuses aanbieden en ik hoop dat u deze wilt aanvaarden. Sorry.

Hello,

First of all I want to thank you for having contacted me. I have a lot of respect for that. I can imagine that it must be very difficult for you to face me in any way. It’s hard for me too.

I can also imagine that you have had to be very scared about what I did in the past period. I would therefore like to say that I am very sorry. I now know how wrong I was and that what I did to you is terrible. You have every right to be angry with me, because this should not have happened to you. It should not have happened to anyone. I will never be able to talk my actions well and that is not what I want either. I just want to apologize and I hope you will accept it. Sorry.
