# Corrigendum: Sincerity Is in the Eye of the Beholder: Using Eye Tracking to Understand How Victims Interpret an Offender's Apology in a Simulation of Victim–Offender Mediation

**DOI:** 10.3389/fpsyg.2020.01943

**Published:** 2020-08-31

**Authors:** Florian Bonensteffen, Sven Zebel, Ellen Giebels

**Affiliations:** Faculty of Behavioral, Management and Social Sciences, Department of Psychology of Conflict, Risk and Safety, University of Twente, Enschede, Netherlands

**Keywords:** victim–offender mediation, apology, sincerity, visual attention, eye tracking, perceived responsibility taking, perceived suffering, offender resocialization attitudes

In the original article, the reference for Zebel, S., Giner-Sorolla, R., and Kamau, C. (2020) was incorrectly written as “Zebel, S., Giner-Sorolla, R., and Kamau, C. (2019). Suffering and responsibility taking inferences explain how victim group members evaluate wrongdoers' expressions of negative feelings. *Eur. J. Soc. Psychol*”. It should be cited as “Zebel et al. (2020), unpublished manuscript^1^”. All occurrences of this citation have been updated, and the incorrect reference has been removed from the reference list.

Additionally, in the original article, the following reference was not cited:

Laxminarayan, M., Lens, K., and Pemberton, A. (2015). “Victim-offender encounters in the Netherland,” in *Victims and Restorative Jusice: Country Reports*, eds D. Bolivar, I. Aertsen, and I. Vanfraechem (Leuven: European Forum for Restorative Justice), 96–133. Available online at: https://www.euforumrj.org/sites/default/files/2019-11/report_victimsandrj-2.pdf.

The citation has now been inserted in the first paragraph of section Taking Into Account Victims' Expected Sincerity and Their Attitudes Towards Resocialization which should read:

Of course, the victims will differ in terms of the expectations and attitudes that forego their engagement in a VOM program (Karliczek et al., 2013; Hansen and Umbreit, 2018). It is inevitable to take into account the victims' prior expectations regarding the sincerity of the apology (henceforth, expected sincerity) when examining the perceived sincerity of the apology as a desired outcome of VOM (Bolívar, 2013; Dhami, 2016). Along the same line, several authors have argued and empirically demonstrated that the motivation to contribute to the offender's restoration or resocialization (by facilitating the offender to make things right and to help the offender go on a better path/not commit crime again) can be an important reason to take part in VOM (Bolívar, 2013; Laxminarayan et al., 2015; Paul, 2015). However, individuals are likely to differ in their a priori attitudes toward programs (such as VOM) that help offenders resocialize (e.g., Maruna and King, 2009).

The citation has also been inserted in the third paragraph of section Conclusion and Future Directions which should read:

For future research, we conclude that eye tracking technology offers substantial potential to gain insight into cognitive and inferential processes that have not been studied before. This paper provides an exploratory approach to apply eye tracking in a simulated victim offender mediation scenario. Considering the fact that VOM programs are applied in a wide range of contexts, more differentiated research is needed toward new directions: In particular, a more process-related research approach will provide more in-depth knowledge about the (un)conscious, emotional processes involved in VOM that might be linked to the beneficial outcomes VOM can produce for victims as well as offenders (Shapland et al., 2007, 2008). This study underlines the importance of such an in-depth approach: receiving and looking at the non-verbal behavior in the upper face of the offender during his apology predicted quite diverging inferences of responsibility taking on the part of the victims, depending on whether they favored or dislike offender resocialization. In turn, these differences in perceived responsibility taking predicted concurrent evaluations of the sincerity of the apology – which is one of the major outcomes of the VOM process for victims (Laxminarayan et al., 2015). These findings suggest that it is important to take into account the victims' a priori orientations toward resocialization in the mediation process as it influences what impact it has for them to look the offender in the eye when making an apology.

The authors apologize for these errors and state that this does not change the scientific conclusions of the article in any way. The original article has been updated.

## Footnotes

^1^Zebel, S., Giner-Sorolla, R., and Kamau, C. (2020). Suffering and responsibility-taking inferences explain how victim group members evaluate wrongdoers' expressions of negative feelings (unpublished manuscript).
