# Supplementary material for: Sincerity Is in the Eye of the Beholder: Using Eye Tracking to Understand How Victims Interpret an Offender’s Apology in a Simulation of Victim–Offender Mediation
Source: Front Psychol. 2020 May 20;11:835. doi: 10.3389/fpsyg.2020.00835 (PMC7251183; doi:10.3389/fpsyg.2020.00835)
Supplement: Supplementary file 1 [file Data_Sheet_1.docx]

*Supplementary material*

This document provides additional measures that are not included in the analysis. In order to allocate this study in the context of contemporary VOM- research examining the beneficial (practical) effects of VOM, such as healing the victim’s emotional harm or reduce the offender’s risk to reoffend, we included additional measures in the research instrument. For the sake of conciseness of this article, these measurements were not included in the final version of the proposed research model.

**1. Fear towards the offense and fear and anger towards the offender before and after mediation**. Zebel (2012) found that taking part in direct, face-to-face mediation is beneficial for victims as it reduces their feelings of fear and anger towards the offender. In this study, we replicated these findings: After participants were instructed to imagine taking the role of a victim in a violent burglary scenario, we measured their feelings of fear after the crime and fear and anger towards the offender. Two scales inquired to what extent participants felt nervous, afraid, panic, insecure and fearful, indicating their perceived fear that is (1) caused by the offense and (2) directed towards the offender two weeks after being victimized (α=.65 resp. α=.72). Anger towards the offender was measured with four items: angry, furious, mad and frustrated, α=.79. These were also used in previous studies (e.g. Jonas - Van Dijk, 2016) and translated from the Dutch language. All measurements were assessed with a five point Likert scale, ranging from strongly disagree to strongly agree. We presented the same scales after participants were exposed to the offender’s apology. Again, all scales had a high reliability with α=.78 (fear towards the offense after mediation), α=.80 (fear towards the offender after VOM) and α=.81(anger towards the offender after VOM). Both expected and perceived sincerity had a high and almost identical negative correlation with fear towards the offense (-.40** and -.38**) and the offender (-.35** and -.31**). Moreover, expected sincerity, all emotional inferences and the perceived sincerity after VOM had a high, significant negative correlation with anger towards the offender after VOM. This indicates that higher (previous) expectations regarding the offender’s sincerity are associated with less anger after mediation but also that higher perceived sincerity after mediation may lead to a stronger decline of anger towards the offender, which is in line with previous studies that accentuate the positive potential of VOM to repair the harm caused by the offender.

**2. Behavioral expectations towards the offender.** We assessed future expectations towards the offender’s behavior with four statements that reflected the likelihood of the offender to become recidivists; two were positively formulated (on a 5 point Likert scale). For example: „I think that the offender is likely to commit a similar crime in the future“. Scale reliability proved to be high with α=.88. This scale was not tested within our research model that primarily focused on the perceived sincerity as an outcome. However we considered this measurement to give us more, interesting a posteriori insights about the victims’ opinion towards the offender. Positive expectations towards the offender’s future behavior (e.g. risk of recidivism) correlated to a significant positive level with the perceived suffering, responsibility taking, empathy and perceived sincerity. When people therefore had positive feelings about the offender in the current mediation scenario, they were also more optimistic regarding changes in the offender’s behavior in a short and long term perspective.

**3. The ability to evaluate the apology and to take the role of the victim.** The purpose of these measurements was to (a) screen the data set (e.g., if participants indicated very low ability to take the victim’s role their data was not used for further analysis) and (b) get to know whether participants answers are likely to reflect ‘real’ crime victims who experienced such a scenario. With a seven point Likert scale we measured how participants rated their own ability to evaluate the apology, represented by the statement „For me, it was easy to make an estimation about the sincerity of the offender’s apology“. Compulsory text entry was attached with space for a short explanation for their choice to give participants space to elaborate on their impressions. The ability to take the role of the victim was assessed with a 10 point Likert scale. No participant had to be excluded from data analysis due to a low score on this scale.

**4. Perceived emotions.** After the stimulus material was shown, we measured which emotions participants noticed to be expressed by the offender. On a five point Likert scale, participants were asked about basic emotions (e.g. sadness, fear) and emotional inferences such as regret and suffering that the offender’s might feel while giving the apology. According to their estimations, the offender’s upper face area gained the most attention, which was in line with the gaze measure.

**5. Perceived regret.** Participants were asked to indicate on a five point Likert scale to what extent they perceive the offender to regret his deed. Two items (one reversed) were presented to measure perceived regret, e.g. „If he could, the offender would make his deed unhappen“. Yet we considered this construct to highly coincide with perceived responsibility taking (see also Robbennolt, 2003 in: Dhami,, 2017) and therefore did not include it in the research model.
